# Late Endocrine Effects after Stem Cell Transplant in a Young Girl with Griscelli Syndrome

**DOI:** 10.1155/2021/9981306

**Published:** 2021-12-27

**Authors:** Shana R. Mencher, William V. Tamborlane, Anisha D. Patel

**Affiliations:** ^1^Children's Hospital at Saint Peter's University, 254 Easton Ave, MOB, 3rd Floor, New Brunswick, NJ 08901, USA; ^2^Yale University School of Medicine, Department of Pediatrics, Section of Endocrinology, 333 Cedar Street LMP 3103, New Haven, CT, USA

## Abstract

*Background*. Griscelli syndrome (GS) is a rare disorder characterized by partial albinism and silver hair with alteration in genes necessary for melanin transport. Type 2 GS is fatal due to severe immunodeficiency without curative stem cell transplant (SCT). Late endocrinopathies are quite common in other disorders after SCT. These complications have not been reported in GS. *Case Presentation*. A 7-year-old female presented for growth failure with a history of GS status post curative SCT and consequently developed graft-versus-host disease (GvHD). She also had a history of eosinophilic enterocolitis, for which she was taking supraphysiologic glucocorticoids for the past year. She presented with severe short stature along with mild hyperthyroxinemia with subsequent diagnosis of Graves' disease, which was treated with methimazole. GH therapy was commenced due to persistent growth failure, with a robust increase in growth parameters. She started spontaneous puberty; however, initial biochemical evaluation revealed hypergonadotropic hypogonadism with undetectable anti-Mullerian hormone (AMH) consistent with low ovarian reserve and premature ovarian failure. *Discussion*. Growth failure was multifactorial due to her inflammatory condition and poor weight gain from multiple underlying illnesses, including hyperthyroidism, as well as chronic supraphysiologic glucocorticoid use. Although hypothyroidism is more commonly seen after SCT, rare cases of hyperthyroidism have been reported. In addition to SCTs, GvHD and GS have been associated with autoimmune conditions. It is important to monitor pubertal progression as the majority of those treated with alkylating agents prior to SCT have pubertal and ovarian failure and remain at risk for premature menopause.

## 1. Introduction

Griscelli syndrome (GS) was first described in 1978, and only approximately 150 cases have been reported in the literature [1, 2]. There are three types of GS, which is an autosomal recessive disease with a phenotype of hypopigmentation and silvery hair due to defects in genes that regulate melanin transport [2].

Our patient was diagnosed with type 2 Griscelli syndrome, which is due to a mutation in RAB27A, an important component of cytotoxic granule exocytosis. This leads to excessive activation of macrophages and ultimately hemophagocytic lymphohistiocytosis [3]. GS type 2 is the only type curative by stem cell transplant (SCT). We present herein a unique presentation highlighting a plethora of endocrinopathies that can be seen after SCT in GS type 2 patients with use of chemotherapeutic agents and chronic supraphysiologic glucocorticoids. Endocrine organs, in particular, are highly susceptible to chemotherapy and irradiation due to a high proportion of growing cells [4]. The rates of various endocrinopathies after SCT can be seen in Table 1. This case also reviews the pathophysiology of growth failure in those with a complex medical history, which is often multifactorial.

## 2. Case Presentation

Our patient, a 7-year-old female of Haitian descent, was diagnosed with GS type 2 during infancy after multiple infections. She received an allogenic SCT (4/6 unrelated umbilical cord transplant) as a toddler, developed graft-versus-host disease (GvHD) at 2 years of age, and presented to our pediatric endocrinology clinic with growth failure at 7 years of age. She received cyclophosphamide, etoposide, busulfan, and rabbit anti-thymoglobulin as part of her conditioning regimen prior to the SCT. She also had a history of atopy and autoimmunity (vitiligo and alopecia) and was taking chronic supraphysiologic doses of glucocorticoids for eosinophilic enterocolitis. On average, she was taking approximately 20–40 mg/m^2^/day (and up to approximately 100 mg/m^2^/day) of hydrocortisone equivalent (using a conversion ratio of 4:1 for prednisolone to hydrocortisone). Unfortunately, she required high doses of prednisolone from ages 6 years 3 months to 7 years 6 months to prevent recurrences of enterocolitis.

Review of systems on presentation for severe growth failure was unremarkable except for intermittent headaches. Physical examination was significant for low weight (−3.22 SDS below the mean), extreme short stature (−6.18 SDS below the mean), and a suboptimal growth velocity of 0.7 inches per year. Vitals were significant for tachycardia to 112 beats/minute but were otherwise unremarkable. She appeared healthy, but much younger than her stated age, with diffuse hypopigmented areas consistent with vitiligo as well as silvery streaks in her hair.

On our evaluation at 7.5 years of age, she had mild hyperthyroxinemia (free thyroxine: 3.3, reference range: 1.0–2.2 ng/dL) with normal thyrotropin of 0.71 mIU/L (reference range: 0.6–5.5 mIU/L). There was a persistent mild elevation in IGF-1 (291 ng/mL, reference range: 155–238 ng/mL) with normal insulin-like growth factor-binding protein 3 (IGFBP-3) and random growth hormone (GH) levels. Additional assessment of thyroid function included elevated thyroid peroxidase (450 IU/mL, reference range: <15 IU/mL) and antithyroglobulin antibodies (>1000 IU/mL) with normal thyroid-stimulating immunoglobulins (34%, reference range: <140%) and thyrotropin binding inhibitory immunoglobulin (<6%, reference range: <16%). Bone age was read as 3 years 6 months, indicating a marked delay of 4 years.

**Figure 1:**
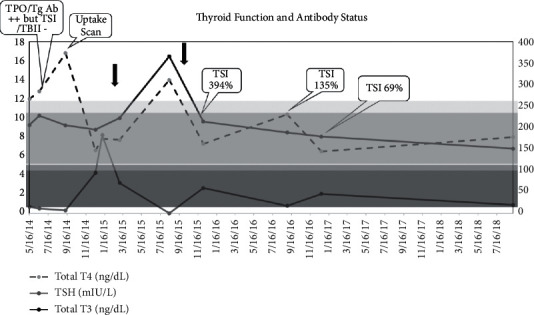
Thyroid course. TSH = thyroid stimulating hormone; TPO = thyroid peroxidase antibody; Tg Ab-thyroglobulin antibody; TBII = thyrotropin binding inhibitory immunoglobulin; TSI = thyroid stimulating immunoglobulins; arrow = glucocorticoid wean.

The patient, who was initially clinically euthyroid, began reporting difficulty sleeping, diaphoresis, and hyperactivity over the next few months, which prompted a thyroid technetium uptake and scan. Intense homogeneous uptake was seen with an elevated 24-hour radioactive iodine uptake at 51% (normal 10–30%), consistent with Graves' disease. She was started on a low dose of methimazole at this time with thyroid course and antibody status seen in Figure 1. As her glucocorticoids were weaned (arrows in Figure 1), levels of triiodothyronine rose and she required an increase in methimazole. These findings were likely related to partial treatment of hyperthyroidism with glucocorticoids. In addition, her thyroid-stimulating immunoglobulins were initially normal but rose approximately 14 months after methimazole treatment was initiated to 394% (reference range: <140%) as shown in Figure 1.

Approximately 1 year after cessation of glucocorticoids and after adequate treatment for her hyperthyroidism, GH treatment was initiated due to persistent growth failure, at a dose of 0.19 mg/kg/week. This was then increased according to growth velocity and IGF-1 level to a max dose of 0.26 mg/kg/week, which resulted in a robust increase in growth rate. At nearly 12 years of age, she was noted to be Tanner stage 3 for thelarche and pubarche with a pubertal growth velocity and normal pubertal progression. Her height was now at the first percentile (−2.25 SDS below the mean) and weight at approximately 10th percentile (−1.13 SDS below the mean). Surprisingly, her bone age remained delayed by about 2.5 years, which was inconsistent with her Tanner staging. Initial evaluation of her pubertal hormones revealed hypergonadotropic hypogonadism (FSH: >86.00, reference range: 0.64–10.98 mIU/mL; LH: 33.25, reference range: 0.04–10.8 mIU/mL; and estradiol: 4.3 pg/mL, AMH: 0.01, reference range: 0.49–3.15 ng/mL), suggesting premature ovarian failure. Despite the initial lack of pubertal progression for 2 years, she attained menarche around 13 years of age, with periods now occurring at regular monthly intervals. Repeat labs (FSH: 9.77 mIU/mL, LH: 9.68 mIU/mL, estradiol: 192 pg/mL, and AMH: 0.03 ng/mL) revealed normal pubertal gonadotropins and estradiol level, although AMH remains nearly undetectable, suggestive of poor ovarian reserve.

## 3. Discussion

It is well known that various endocrinopathies have been demonstrated after SCT due to the conditioning regimen, underlying condition, and subsequent complications, especially GvHD [1–5] (Table 1). In our patient, Graves' disease was the first endocrinopathy treated and is rather rare after SCT [5] (Table 1). It may have been due to immune dysregulation and reconstitution in the setting of GvHD [6]. In addition, it is prudent to consider autoimmune dysregulation from her underlying disorder. Despite the underlying cause, this case illustrates the need to carefully screen for autoimmune conditions in similar populations.

Our patient also manifested severe growth failure and short stature, despite persistent, modest elevations in IGF-1 levels and normal random circulating concentrations of growth hormone and IGFBP-3. Chronic inflammation from underlying illness and SCT leads to dysregulation of the GH-IGF-1 axis at both the central and peripheral level ultimately leading to GH and IGF-1 resistance at the growth plate, which may explain her elevated IGF-1 level [7].

As in our patient, abnormally low growth velocity and markedly delayed bone age may be observed in young patients being treated with chronic supraphysiologic glucocorticoids [8].

As in other cases, glucocorticoids likely contributed to her growth failure by blunting pulsatile GH release through augmentation of hypothalamic somatostatin tone [7]. Glucocorticoids also downregulate hepatic and growth plate GH receptor expression; inhibit growth factor and osteoblast activity; accelerate chondrocyte apoptosis; and suppress collagen synthesis and adrenal androgen production [7]. Nonetheless, our patient manifested severe short stature even after discontinuation of glucocorticoids. In addition, poor weight gain due to chronic colitis likely played a significant role in growth failure which was exacerbated in the setting of hyperthyroidism.

Finally, her initial hypergonadotropic hypogonadism can be attributed to her conditioning treatment with the alkylating agents busulfan and cyclophosphamide prior to her SCT. However, more recent labs may indicate some residual ovarian function and it is reassuring that she has progressed through puberty spontaneously. Nonetheless, it is essential to continue to monitor as only 10–14% of females after SCT have shown recovery of ovarian function and she remains at risk for early menopause [9].

## 4. Conclusion

There are many late endocrine effects associated with SCT and its complications, especially graft-versus-host disease. Our patient illustrates the benefits of growth hormone treatment of severe short stature as well as the need to closely monitor for thyroid dysfunction and ovarian failure. Despite its curative nature for Griscelli syndrome type 2, SCT and its complications as well as the underlying illness need to be considered when screening for various endocrinopathies, in particular autoimmune disease.

## Figures and Tables

**Table 1:** Endocrinopathies after stem cell transplant.

Endocrinopathies	Incidence
Thyroid dysfunction (i) Autoimmune thyroid disease (ii) Hyperthyroidism	30% in pediatric patients, 15% in adults^1^ (i) 2.9% after allogenic SCT, 4% after autologous SCT^2^ (ii) 1.3% in pediatric study (75% with autoimmunity)^3^
Growth impairment	20–85% in pediatric patients^4^
Ovarian failure	65–84% in pediatric patients^1^
Pubertal failure	57% of prepubescent females^1^

^1^Dvorak CC, Gracia CR, Sanders JE, et al. NCI, NHLBI/PBMTC first international conference on late effects after pediatric hematopoietic cell transplantation: endocrine challenges-thyroid dysfunction, growth impairment, bone health,and reproductive risks. *Bioi Blood Marrow Transplant.* 2011; 12:1725–38. ^2^Au, WY, Lie, AK, Kung AW, et al. Autoimmune thyroid dysfunction after hematopoietic stem cell transplantation. *Bone Marrow Transplant.* 2005; 35 (4):383–8. ^3^SagE, Gont; N, Alika ifoglu A, et al. Hyperthyroidism after allogeneic hematopoietic stem cell transplantation: A Report of Four Cases. *J Clin Res Pediatr Endocrinol.* 2015; 4:349–54.0rio F, et al. *The Scientific World Journal.* 2014. ^4^Orio F, Muscogiuri G, Palomba S, et al. Endocrinopathies after allogeneic and autologous transplantation of hematopoietic stem cells.*Sci World J.* 2014; 2014:1–13.

## Data Availability

Data were obtained directly from the patient and her medical records.
